# Secondary use of radiological imaging data: Vanderbilt’s ImageVU approach

**DOI:** 10.1016/j.jbi.2025.104905

**Published:** 2025-09-10

**Authors:** David S. Smith, Karthik Ramadass, Laura Jones, Jennifer Morse, Daniel Fabbri, Joseph R. Coco, Shunxing Bao, Melissa Basford, Peter J. Embi, Reed A. Omary, John C. Gore, Jill M. Pulley, Bennett A. Landman

**Affiliations:** a Vanderbit University Institute of Imaging Science, Vanderbilt University Medical Center, Nashville, TN, USA; b Department of Radiology and Radiological Sciences, Vanderbilt University Medical Center, Nashville, TN, USA; c Department of Electrical and Computer Engineering, Vanderbilt University, Nashville, TN, USA; d Vanderbilt Institute for Clinical and Translational Research (VICTR), Vanderbilt University Medical Center, Nashville, TN, USA; e Department of Biomedical Informatics, Vanderbilt University Medical Center, Nashville, TN, USA; f Department of Medicine, Vanderbilt University Medical Center, Nashville, TN, USA; g Department of Biomedical Engineering, Vanderbilt University, Nashville, TN, USA; h Greenwell Project, Nashville, TN, USA

**Keywords:** Biomedical imaging informatics, Secondary use of radiological data, Research imaging infrastructure, Research data warehouse, DICOM, De-identification, Protected health information, Radiomics, Translational research

## Abstract

**Objective:**

To develop ImageVU, a scalable research imaging infrastructure that integrates clinical imaging data with metadata-driven cohort discovery, enabling secure, efficient, and regulatory-compliant access to imaging for secondary and opportunistic research use. This manuscript presents a detailed description of ImageVU’s key components and lessons learned to assist other institutions in developing similar research imaging services and infrastructure.

**Methods:**

ImageVU was designed to support the secondary use of radiological imaging data through a dedicated research imaging store. The system comprises four interconnected components: a Research PACS, an Ad Hoc Backfill Host, Cloud Storage System, and a De-Identification System. Imaging metadata are extracted and stored in the Research Derivative (RD), an identified clinical data repository, and the Synthetic Derivative (SD), a de-identified research data repository, with access facilitated through the RD Discover web portal. Researchers interact with the system via structured metadata queries and multiple data delivery options, including web-based viewing, bulk downloads, and dataset preparation for high-performance computing environments.

**Results:**

The integration of metadata-driven search capabilities has streamlined cohort discovery and improved imaging data accessibility. As of December 2024, ImageVU has processed 12.9 million MRI and CT series from 1.36 million studies across 453,403 patients. The system has supported 75 project requests, delivering over 50 TB of imaging data to 55 investigators, leading to 66 published research papers.

**Conclusion:**

ImageVU demonstrates a scalable and efficient approach for integrating clinical imaging into research workflows. By combining institutional data infrastructure with cloud-based storage and metadata-driven cohort identification, the platform enables secure and compliant access to imaging for translational research.

## 1. Introduction

Secondary data use has become an essential aspect of modern learning healthcare systems; patient data drives both continuous improvement through operational quality protocols and innovation in research to solve real-world challenges in care delivery [1]. Identified research repositories present key opportunities for linking across data sources, augmenting patient data collected under direct consent, and enabling flexible study coding—but retaining identifiable information introduces potential risk. Meanwhile, de-identified repositories allow linkage to sensitive data (such as genetic information) with reduced risk to patient privacy. Vanderbilt University Medical Center (VUMC) has pioneered a dual approach by maintaining an identified copy of secondary data in the Research Derivative (RD) and a de-identified (including date-shifted) copy in the Synthetic Derivative (SD), which serves as the recommended data source when identifiers are not needed [2]. To support genetic research, VUMC also created BioVU, a de-identified DNA biobank [3]. These efforts at secondary data use have evolved into successful national initiatives that integrate both tabular and unstructured electronic health data with genomic characterization [4].

Despite the growing success of secondary use in structured EHR and genomic data, radiological imaging remains an underutilized resource in translational research. Clinical imaging holds rich phenotypic and structural information that complements traditional data sources, offering insights into disease presentation, progression, and treatment response [5]. However, due to its complex formats (e.g., DICOM), large file sizes, and historically siloed storage, imaging data have not been as widely reused as tabular or genetic data in research pipelines [6].

This under-use is significant because imaging can uncover clinical attributes — such as body composition, bone density, and liver fat — that are often missing from structured records, and can be derived from existing scans using opportunistic imaging approaches [7]. For example, waist circumference is rarely recorded in electronic health records (EHR), either due to workflow burden or lack of clinical emphasis, but it can be accurately derived from abdominal CT scans using automated processing. This allows researchers to estimate body composition and metabolic risk without additional patient interaction [6]. Such latent information underscores the potential of imaging data to enrich secondary use, especially in disease characterization, risk stratification, and precision medicine [8].

Leveraging clinical imaging data for research presents institutional and operational challenges. Imaging systems are typically optimized for clinical care, not for large-scale data access, making it difficult to repurpose data for research without disrupting clinical workflows [9]. Imaging studies are stored across fragmented systems, and retrieving them at scale often requires manual processes or ad hoc technical support. Furthermore, research access must navigate issues of privacy, governance, and data security, especially when dealing with identified imaging or protected health information (PHI) [10]. Our work aims to lower these barriers by streamlining access and enabling broader use of radiographic data—even for researchers without domain-specific imaging expertise—thereby helping to democratize imaging-based research.

With the growing volume of imaging data, there is a clear need for infrastructure that supports routine secondary use in a way that is scalable, compliant, and aligned with institutional priorities. To address these gaps, we developed ImageVU, the VUMC research imaging archive—a purpose-built platform that facilitates seamless access to clinical imaging for research, while minimizing burden on clinical operations and IT systems.

### Significance.

A structured summary of the significance of this work is presented in Table 1.

## 2. Methods

The Methods section introduces the design and implementation of ImageVU, including its system architecture, core components, integration with VUMC’s research data infrastructure, and the workflow for researcher access and data delivery.

### 2.1. Research landscape

The Vanderbilt Institute for Clinical and Translational Research (VICTR) [11] provides the institutional foundation for clinical and translational research at VUMC. Supported by the VUMC Office of Research and the NIH-sponsored Clinical and Translational Science Award (CTSA), VICTR offers tools and services to accelerate innovation and improve the quality of research, publications, grant writing, and training for future clinicians and scientists.

As part of VICTR, the Office of Research Informatics (ORI) comprises a team of engineers, data scientists, and product managers dedicated to building a secure and flexible data ecosystem to support a broad range of researchers. ORI develops and maintains tools such as REDCap (Research Electronic Data Capture) [12], along with other secure platforms that facilitate data collection, consent tracking, and project management (e.g., STARBRITE [13]). ORI also enables researchers to apply for and receive preliminary project funding through streamlined digital workflows.

In general, ORI oversees the secondary use of clinical data and manages a centralized collection of research tools and services, offered at affordable cost through a fee-for-service model. Billing is processed via the Core Ordering and Reporting Enterprise System (CORES) [14], a centralized platform that supports equitable access and long-term sustainability. This funding model addresses two common challenges observed at other institutions: lack of scalable access and lack of sustainable operational support [15].

VICTR also houses the Vanderbilt Data Warehouse team, which manages two foundational research repositories: the RD and the SD, which Both are accessible through user-facing applications, a fee-for-service core, or direct access (see Sections 2.4 and 2.5). ImageVU extends VICTR’s existing data infrastructure, leveraging established tools, governance, and technical teams to enable imaging data reuse within the institutional research ecosystem.

### 2.2. Clinical enterprise

Clinical imaging acquisition and organization are primarily designed to meet diagnostic and long-term archival needs. These objectives, driven by clinical and business operations, are often misaligned with the demands of research workflows. At VUMC, the central clinical imaging platform is Sectra,^1^ a modern picture archiving and communication system (PACS) [16]. Within VUMC Radiology, Sectra supports diagnostic interpretation, image archiving, provider communication, and integration with the Radiology Information System (RIS) [17] for administrative and billing purposes.

Although Sectra is optimized for rapid, reliable clinical operations, it is not well suited for large-scale historical data queries and transfers—known in DICOM terminology as C-FIND and C-MOVE operations [18]. Clinical PACS environments typically operate near full capacity to support day-to-day care. Large research data pulls can match or exceed clinical imaging volumes, and attempting to serve both clinical and research needs from the same system can lead to performance degradation, system contention, and delays in both workflows [19].

In addition, Radiology IT staff are generally funded through clinical operations and are not resourced to support research data requests, leaving limited institutional capacity to accommodate large-scale research access. Without a dedicated infrastructure, imaging researchers are often left to manually retrieve studies through clinical PACS workstations—a process that is labor-intensive and error-prone.

To address these limitations, the ImageVU team established a real-time routing pipeline that sends all clinical CT, MR, and PET imaging studies to a secure research-facing archive. A dedicated router duplicates incoming imaging traffic and forwards it to the ImageVU Research PACS. The only modifications required by clinical IT were to add the Research PACS as a destination for these imaging modalities and adjust firewall permissions. This passive duplication approach ensures that ImageVU receives a complete stream of imaging data without disrupting clinical workflows or requiring continuous IT intervention.

### 2.3. ImageVU architecture overview

ImageVU is designed for scalability and regulatory compliance while minimizing disruption to clinical operations. By operating independently of clinical systems, it enables high-throughput research access without affecting clinical performance. As a secondary-use platform, ImageVU relies on external systems such as the clinical PACS, hospital networks, and cloud infrastructure. These dependencies present challenges related to uptime, data availability, and system access, which are mitigated through modular design and automation.

ImageVU consists of four primary interrelated subsystems, each built using open-source software for flexible, standards-compliant manipulation of DICOM imaging data:

- Research PACS: Receives, indexes, and temporarily archives clinical imaging studies in a secure, research-focused environment.
- Ad Hoc Backfill Host: Retrieves historical or missed imaging data not captured in the real-time pipeline, enabling retrospective cohort completion.
- Cloud Storage System: Offers long-term, encrypted storage and supports high-throughput delivery of imaging data for downstream use.
- De-Identification System: Applies either investigator-defined rules or SD–compatible mappings (e.g., replacing Person IDs with Study IDs, applying date shifting) for project-specific privacy compliance.

**Fig. 1.**
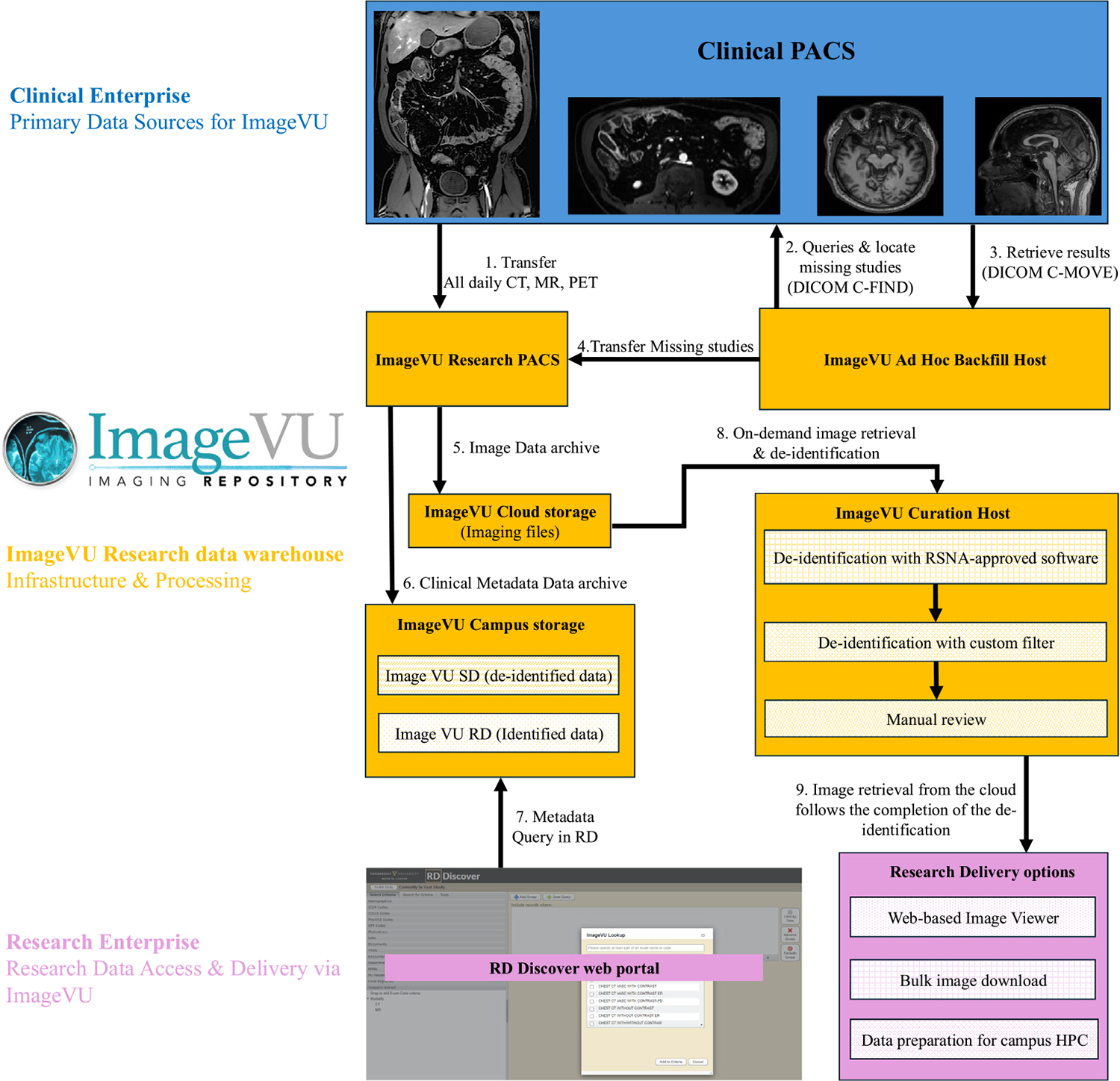
Host and network architecture of the ImageVU infrastructure service. The clinical radiology PACS feeds downstream research servers that do not have permission to send studies to the clinical PACS. A primary nightly stream of all CT, MR, and PET studies feeds the research PACS, while missing modalities and studies prior to the initiation of the nightly copy are backfilled on an ad hoc basis using a separate server that can send DICOM C-FIND queries to the clinical PACS and retrieve studies via DICOM C-MOVE. Imaging files are stored in Google Cloud Platform (GCP) in their identified form. Clinical metadata is archived in ImageVU Campus Storage, where ImageVU RD (identified metadata) and ImageVU SD (de-identified metadata) are maintained, for further cloud-based indexing. When researchers request access, the ImageVU Curation Host retrieves the requested imaging from ImageVU Cloud Storage and performs de-identification (RSNA CTP, custom filters, and manual review) before releasing the data for research use. The internal research servers coordinate storage, preparation, and delivery of research studies. Researchers use RD Discover to query metadata before retrieving imaging data through web-based viewers, bulk downloads, or high-performance computing (HPC) processing.

These components are connected through custom interface layers that coordinate data flow and ensure resilience. Imaging metadata extracted by ImageVU are integrated into VUMC’s existing research data warehouses—the RD and SD—as dedicated imaging views, referred to as ImageVU RD and ImageVU SD. These views support metadata-driven cohort discovery and linkage with other clinical resources. As illustrated in Fig. 1, ImageVU connects to the clinical PACS in a manner that avoids overloading production systems, while ensuring regular metadata updates to RD and SD. Researchers identify cohorts using metadata stored in the RD and request access to corresponding imaging data, which are delivered via cloud-based services and supported through VUMC’s Integrated Data Access and Services Core (IDASC), which supports web-based viewing, bulk download, and high-performance computing workflows.

#### 2.3.1. ImageVU research PACS

The ImageVU Research PACS runs on a physical CentOS Linux server using DCM4CHEE [20], housed in the VUMC data center in the same secure server room as the Sectra clinical PACS. The clinical Sectra and the research receive node are on the same VLAN and physical LAN to prevent potential issues such as packet sniffing during clinical data transmission. However, once data are received by the research node, all storage and transmission processes are fully encrypted, both in transit and at rest. The cloud architecture supporting the research PACS operates within the institution’s Business Associate Agreement (BAA) and is a covered entity under institutional agreements. Importantly, the anonymization processing and cloud infrastructure are isolated from the clinical PACS to ensure computational separation and to maintain clinical service priority. Thus, this co-location provides enhanced physical security and streamlined network access.

Several free and open-source PACS solutions, such as Orthanc [21], Dicoogle [22], and Conquest DICOM Server [23], are available and widely used in research environments. In this project, we selected DCM4CHEE as our research PACS platform primarily due to its open-source availability and its proven enterprise-level maturity. While we did not perform a full comparative scalability analysis across plat-forms, this remains an important area for future work to systematically evaluate the performance, scalability, and interoperability of various open-source PACS solutions in research settings.

The primary storage consists of a 5.5 TB solid-state RAID array, offering approximately 48 h of buffer capacity in case of interruptions between local and remote storage destinations. A local firewall and port router sits between the VUMC network and the Research PACS, allowing dynamic redirection of traffic for server maintenance without disrupting upstream systems.

To support failover and disaster recovery, a redundant physical server is maintained in the same location. This live backup enables seamless switching during downtime and provides capacity for warm recovery of the Research PACS in the event of system failure.

A background process monitors the local archive for new imaging sessions that remain unmodified for at least one hour. Once eligible, sessions are transferred to the ImageVU cloud storage (see Section 2.3.3), and local storage is released. The PACS database is backed up to the cloud nightly to ensure redundancy. In parallel, the imaging metadata needed for cloud-based indexing is extracted and shared with the VICTR Data Warehouse Team. This partial metadata, which may still include identifiers (e.g., MRN, accession number, modality, and series details), is integrated into structured tables in RD and SD (see Sections 2.3.5 & 2.3.6). Within RD/SD, the metadata undergoes their own robust de-identification procedures (see Section 2.3.4), which can later be used as phenotype criteria to assess study feasibility and define study cohorts. The complete imaging metadata is preserved in the Research PACS without modification.

#### 2.3.2. ImageVU Ad Hoc Backfill server

ImageVU contains a nearly comprehensive collection of MR, CT, and PET imaging studies acquired at VUMC from 2007 to the present. However, some gaps exist due to scanner outages, system downtimes, or network disruptions during image transmission. Because clinical images are routed to the ImageVU Research PACS in real time at the point of acquisition, a separate mechanism is required to recover missing studies and fulfill historical data requests.

To address this, ImageVU employs a dedicated backfill process that operates independently from the primary routing pipeline. This slower, asynchronous process retrieves requested historical studies directly from the clinical PACS and transfers them to the ImageVU Research PACS. The system is deliberately throttled to avoid interfering with clinical operations. Retrieved images are first stored locally on the backfill host before being routed to their final destination in the research archive.

#### 2.3.3. ImageVU cloud storage

ImageVU utilizes a commercial cloud provider, Google Cloud Platform (GCP) [24], to manage encrypted-in-transit and encrypted-at-rest storage of research imaging data. A secure cloud storage bucket is maintained for this purpose, with access and encryption policies governed by the ORI in alignment with VUMC enterprise security standards.

All DICOM files within a session are bundled into a single TAR archive and compressed using BZIP2, using the full unique pathname from the Research PACS as the storage key. This approach is selected to improve storage efficiency and reduce latency during cloud operations. While compression may reduce transfer size, the main design priority is efficient long-term storage in a write-once, read-many system. The original DICOM transfer syntax, including JPEG Lossless if applicable, is fully preserved within the bundle. Cloud storage encryption is applied at the bucket level using native cloud provider encryption services, ensuring data are encrypted both at rest and in transit. The DICOM Storage SCP is used for initial data ingestion, after which files are archived in the compressed bundles without altering the original DICOM encoding. Data retrieval is performed by accessing the archived bundle and extracting the relevant DICOM files as needed. This design supports efficient session-level retrieval through single input/output operations.

When a session is pushed from the Research PACS to the cloud, the system first checks for an existing entry. If a prior version is found, it is downloaded, merged with new content, and re-uploaded; otherwise, the session is uploaded as new. To retrieve a session, the complete archive is downloaded in its entirety. Any modifications must be performed locally, and the updated session must then be re-uploaded to overwrite the previous version.

#### 2.3.4. ImageVU curation host

The ImageVU Curation Host is a local server responsible for de-identifying imaging data only when an access request is made. It manages the de-identification workflow for datasets pulled from the ImageVU Cloud Storage after cohort selection is complete.

The de-identification pipeline follows a multi-stage process to ensure all PHI is removed before research access. In the first pass, images are processed using the open-source RSNA Clinical Trial Processor (CTP), developed by the Radiological Society of North America as part of the Medical Imaging Resource Center (MIRC) framework [5], which removes standard DICOM headers and overlays containing PHI. This step typically results in a residual ID rate of approximately 2%. The Residual ID rate, defined as the proportion of remaining PHI found after automated de-identification, was used to assess de-identification quality. SD-style anonymization refers to the de-identification process used in VUMC SD, which includes removing direct identifiers, shifting dates, hashing IDs, and scrubbing PHI from clinical text to enable research while protecting patient privacy.

In the second pass, a custom filter developed by the ImageVU team is applied to further exclude image types not reliably de-identified by CTP anonymizer module. These include screenshots, scans of reports, prior reports embedded in 3D reconstructions, and other atypical formats. This step reduces the residual ID rate to approximately 1%, and the filter is continually refined across additional projects.

The third pass is conducted through manual review by ImageVU Imaging staff. Manual review is performed for non-VUMC principal investigators or for VUMC investigators sharing data under a Data Use Agreement, to confirm the absence of any residual PHI. Specifically, manual de-identification are reviewed using a custom DICOM viewer built with Pybossa [25,26], which converts DICOM images into video files to streamline manual quality assurance. These images are temporarily stored and finalized only after passing manual review to ensure all project-specific de-identification standards are met.

The final output is prepared and delivered according to the level of de-identification approved by the institutional review board(IRB), ranging from SD-style anonymization to retention of identifiers for identified studies.

#### 2.3.5. The identified metadata layer (ImageVU RD)

ImageVU RD is a dedicated imaging metadata view within the larger VUMC RD, the institution’s identified clinical data warehouse. While the RD contains a wide range of clinical data types, including diagnoses, laboratory results, procedures, medications, and encounter records, ImageVU RD specifically focuses on structured metadata extracted from imaging studies. These metadata include DICOM-level fields such as modality, study date, accession number, and body region, derived from imaging studies stored in the ImageVU Research PACS. Metadata updates follow the established institutional refresh schedule, which occurs monthly.

#### 2.3.6. The de-identified metadata layer (ImageVU SD)

ImageVU SD is the de-identified imaging metadata layer integrated into the VUMC SD, the institution’s de-identified clinical data repository. It includes metadata from all radiologic imaging studies housed in the ImageVU Cloud Storage, as well as completed studies recorded in the EPIC-derived Clarity imaging orders table, which provides structured information on modality, scheduling, and order status. These metadata link imaging studies to patient encounters and support cohort discovery within the ImageVU ecosystem. While the SD layer itself contains no PHI, the metadata may reference imaging studies that have not yet been manually de-identified at the image level. In such cases, access to the corresponding image files requires additional review and de-identification prior to release.

Importantly, we do not restrict the SD imaging metadata to only those studies that have been de-identified. Instead, metadata from imaging studies are curated based on project-specific research cohorts (as described in Section 2.3.4), with key non-identifiable fields derived from DICOM headers. The metadata is updated on a semiannual basis, consistent with the SD refresh schedule.

Projects and cohorts in ImageVU SD are organized around patient Global Registration Identifier for Donors (GRIDs), which serve as the linkage between cohort definitions and imaging metadata. Cohort-level metadata is extracted and stored in structured legacy tables (such as Table 2) and ImageVU Cloud Storage study metadata table (Table 3), which capture key study attributes like modality, study date, and accession number.

### 2.4. Research access and data workflow

Researchers seeking to use ImageVU data must follow a structured approval and access process aligned with VUMC institutional policies. Access to ImageVU RD requires IRB approval or a non-research determination, depending on project scope. For ImageVU SD, an IRB determination of non-human subjects research is required. Because ImageVU SD is part of the de-identified VUMC’s SD, it is limited to IRB-approved research projects. In contrast, non-research activities—such as quality improvement or operational initiatives—must use identified data available in ImageVU RD, as ImageVU SD is restricted to IRB-approved research use only. Template IRB language is available to support protocol preparation.

SD Discover is a web-based cohort discovery tool that provides an interface like RD Discover, supporting structured, drag-and-drop query building on Observational Medical Outcomes Partnership (OMOP)-cleaned data. The key difference is that SD Discover operates on the de-identified, date-shifted SD dataset, while RD Discover uses the identified RD dataset. Unlike RD Discover, SD Discover does not include image-related metadata.

Once regulatory approvals are in place, researchers submit an electronic ImageVU access request. For de-identified projects, cohorts are currently defined by the ImageVU team, since ImageVU metadata is not yet integrated with SD Discover. Once integration is complete, SD Discover will allow researchers to define imaging cohorts directly, similar to RD Discover. Until then, only RD Discover is available for direct cohort definition.

**Fig. 2.**
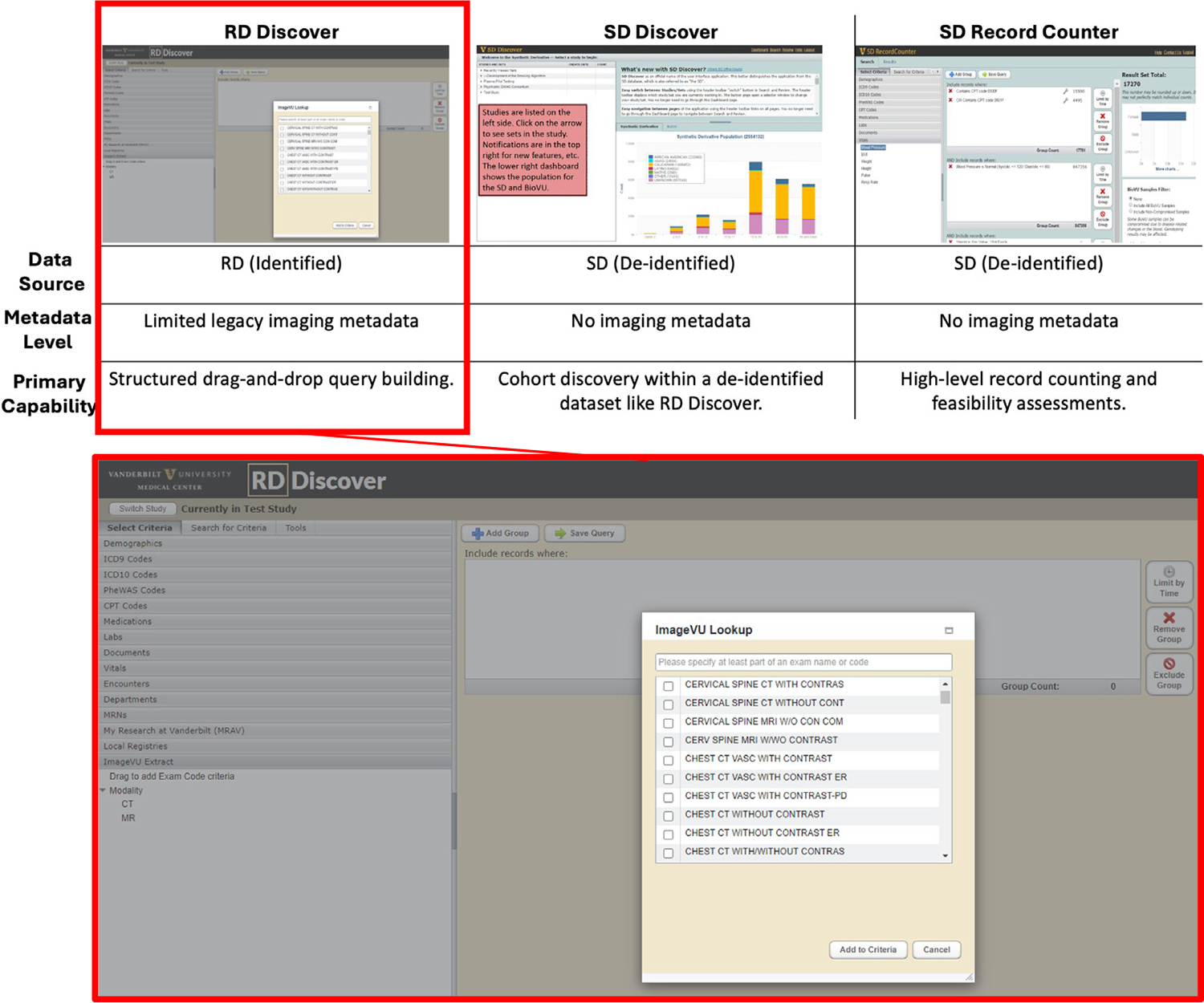
The figure summarizes the primary data sources, metadata levels, and key capabilities of RD Discover, SD Discover, and SD Record Counter. Screenshots are provided for each tool to visually support the description of their distinct roles within the ImageVU system. Zoomed-in view of RD Discover’s interface to improve readability. Specifically, investigators submit data access requests in RD Discover, and the ImageVU team assists in defining phenotype criteria using structured clinical and imaging metadata. Identified studies can be also queried using RD Discover, which allows users to filter datasets by modality, session descriptions, data ranged, and clinical variables, supporting complex AND/OR logic for cohort selection.

For identified projects, researchers utilize RD Discover, a self-service query portal that enables filtering based on clinical and imaging metadata (see Fig. 2). RD Discover supports complex cohort definitions using logical operations (e.g., AND/OR) across demographic, clinical, and imaging criteria. Available imaging metadata fields include modality (e.g., CT, MRI), body region, study date, and session description keywords. Temporal filters such as date ranges may also be applied to refine cohort selection.

Researchers can define cohorts using either SD Discover or RD Discover. However, imaging retrieval requires a backend process managed by the ImageVU team, as imaging data are not directly accessible through the Discover platforms and remain stored in separate PACS systems. For SD-based workflows, researchers typically submit GRID lists, which the ImageVU technical team securely maps to accession numbers to locate the corresponding imaging studies. These studies are then de-identified and made available for research use.

ImageVU imaging metadata is not yet integrated into SD Discover or SD Record Counter due to the technical challenges of mapping unstructured imaging data to standardized formats in a scalable and de-identified framework. This integration is actively in progress and will be supported in future system updates.

Currently, only limited legacy metadata is incorporated into RD Discover. Imaging metadata from the current ImageVU Cloud Storage GCP-managed metadata is not yet integrated. At present, RD Discover primarily supports basic filtering, such as identifying the presence of CT or MRI based on series-level metadata. Full imaging metadata integration remains in progress.

The backend system uses a Postgres database [27] to store and query imaging metadata. Searches are currently executed using structured SQL queries. While search-engine-based technologies (e.g., Elasticsearch) are not yet implemented, future development may consider their integration to improve performance for large-scale federated queries.

Moreover, the current ImageVU platform focuses on institutional-scale imaging reuse, supporting DICOM-level queries via a Postgres-based [27] metadata index and pydicom [28] for header processing. While distributed and federated imaging infrastructures are not currently implemented, future work may explore integration with frameworks such as XNAT federation [29], Dicoogle’s distributed architecture [22], and OHIF-based multi-site viewers [30] to enable scalable, cross-institutional image management and querying.

### 2.5. Research support services and biostatistical guidance

In addition to infrastructure and data access, the ImageVU team collaborates with the Vanderbilt IDASC, a fee-for-service research informatics unit. The IDASC team performs study-specific cohort construction using clinical and imaging metadata from the VUMC RD and SD, including the ImageVU metadata layers. Services include phenotype definition, case-control matching, creation of customized data dictionaries, and extraction of clinical variables such as demographics, laboratory results, diagnoses, and procedures.

ImageVU supports a range of downstream research workflows, from manual review and image annotation to automated image processing and biomarker extraction. The ImageVU team also provides biostatistical input for cohort refinement and outcome specification, and offers technical consultation to support integration of imaging data into broader computational pipelines.

### 2.6. Summary of open-source tools utilized in ImageVU

Table 4 summarizes the key open-source tools integrated into the ImageVU platform to support imaging ingestion, processing, automation, and quality assurance workflows.

## 3. Value proposition and impact

**Fig. 3.**
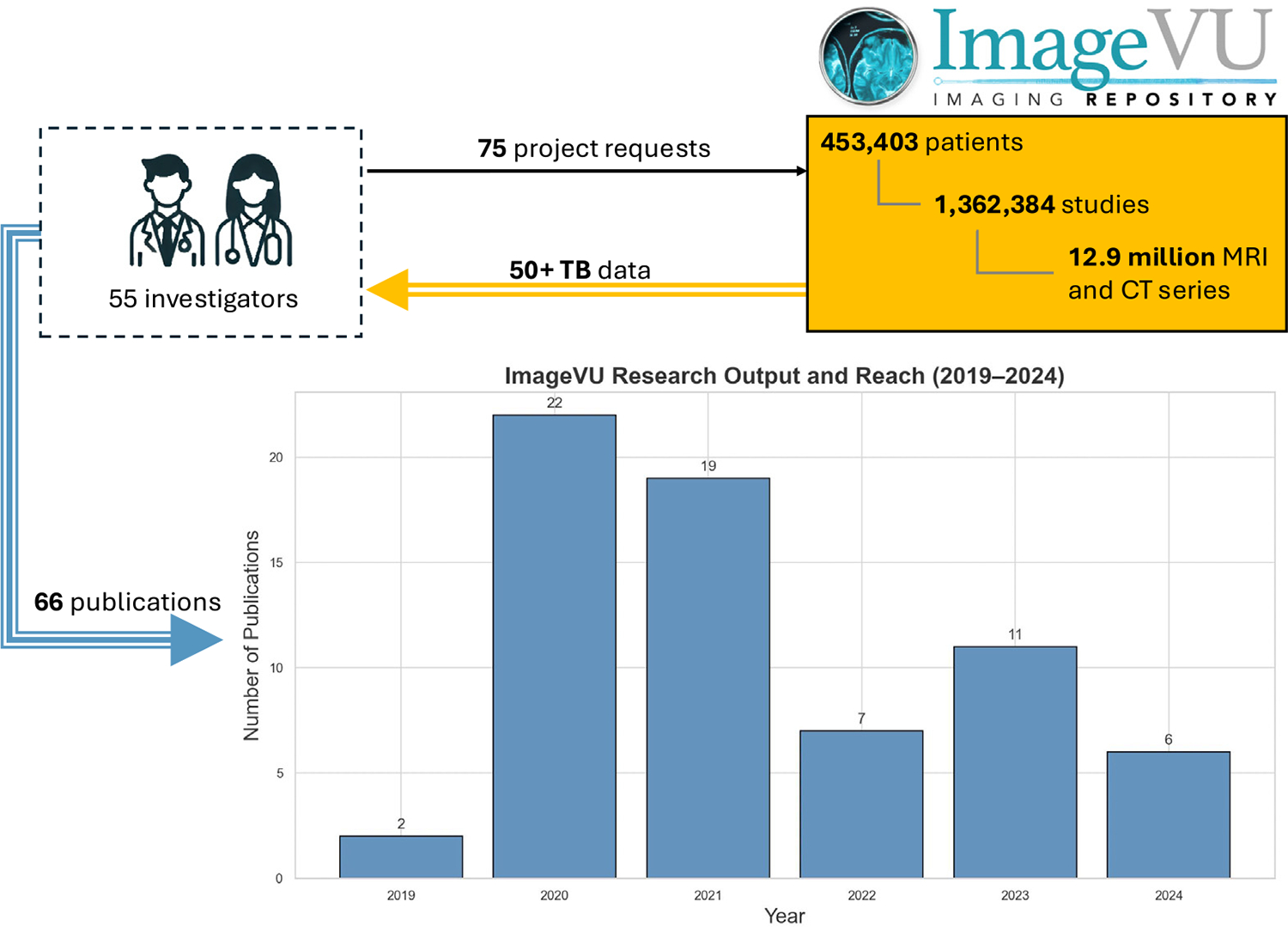
Research utilization and output of ImageVU from 2019 to 2024. Fifty-five investigators initiated 75 project requests involving 453,403 patients, 1,362,384 studies, and 12.9 million MRI and CT series. Over 50 TB of imaging data were delivered, contributing to 66 research publications. The bar chart shows the yearly distribution of publications resulting from ImageVU-enabled research.

Since its inception, ImageVU has demonstrated strong institutional impact. As of December 2024, the platform has supported 75 project requests, delivering over 50 TB of imaging data to 55 investigators and contributing to 66 published research papers (Fig. 3). The de-identified imaging dataset currently contains over 12.9 million MRI and CT series from 1,362,384 studies involving 453,403 unique patients at VUMC.

### 3.1. Improved compliance and informatics guidance

A centralized research imaging infrastructure improves regulatory compliance and standardizes data handling practices. ImageVU ensures consistent image de-identification workflows, reduces the number of individuals interacting with PHI, and enforces centralized security controls. Additionally, the ImageVU team provides expertise in imaging phenotyping and curation. This guidance is especially valuable for researchers with limited experience in imaging analysis. Early involvement improves data quality and supports more robust project development.

### 3.2. Scalable backend infrastructure

ImageVU supports automated image processing pipelines capable of batch analysis of newly acquired imaging data. These pipelines allow researchers to receive processed results without requiring dedicated HPC environment or technical expertise, and reduce turnaround time for imaging analysis at scale.

The data replication process within the ImageVU architecture depends on the specific IRB protocol. For approved studies, imaging data are copied from the ImageVU Cloud Storage (the original, identified imaging data format) to an ACCRE-managed research partition, where de-identification is performed. This is a network-based data transfer, not a physical copy. The de-identified data are then made accessible for research use.

### 3.3. Cost efficiency through shared resources

By centralizing imaging storage and computation, ImageVU enables high reuse of infrastructure, lowering the per-study cost of research imaging. This model supports cost-effective access for early-stage projects and investigators with limited funding, such as pilot studies and trainee-led research.

### 3.4. Support for rapid prototyping

The infrastructure enables quick turnaround on small cohort curation, de-identification, and delivery, supporting fast iteration on pilot studies. This capability is particularly important for research involving rare diseases with limited populations or studies requiring proof-of-concept results prior to scaling.

### 3.5. Institutional research advantage

A robust research imaging platform strengthens institutional competitiveness in securing grants and contracts. ImageVU also supports faculty recruitment and enhances institutional visibility by offering sustainable infrastructure for high-impact, imaging-enabled translational research.

## 4. Lessons learned

The design of ImageVU closely followed the model developed for VUMC’s core secondary data use infrastructure, particularly BioVU [2]. Initial development efforts created separate access systems for clinical and imaging data, similar to the approach used for BioVU, VUMC’s large-scale de-identified DNA biobank [3]. Subsequent changes to the NIH Common Rule [31] spurred a shift toward unified operations and opt-in models for secondary data use.

Today, ImageVU is integrated as an imaging data domain within the broader VUMC RD and SD ecosystems. This integration simplifies investigator access, streamlines IRB documentation, and ensures consistent handling of sensitive information through a single point of governance.

### 4.1. Avoiding clinical impacts while leveraging clinical enterprise data

A core technical design criterion for ImageVU was to avoid disrupting clinical performance while leveraging the clinical imaging archive. Radiology’s primary PACS systems are optimized for limited, concurrent access by clinical users. High-throughput research activity, such as batch downloads or loading thousands of studies, can overwhelm compute, network, and storage resources. This results in degraded performance for frontline clinical users. In prior instances, rapid automated batch pulls or excessive parallel access from research systems caused measurable slowdowns in clinical operations.

To mitigate this risk, ImageVU was designed around a dedicated Research PACS that stores a shadow copy of clinical imaging data. With support from Radiology IT, data routing rules were implemented to transmit studies to the Research PACS in real time upon ingestion into the clinical PACS. This routing occurs within the private institutional network and does not interfere with clinical workflows.

System stability was another critical concern. Interruptions in the data feed—due to maintenance or downtime—could cause backlog in the clinical PACS worklist and generate tasks requiring manual intervention by clinical IT staff. To reduce this operational burden, the ImageVU Research PACS was built on the open-source DCM4CHEE platform, selected for its stability and extensibility after evaluating several alternatives, including Big Data and NoSQL-based architectures. To support maintenance and failover, the Research PACS sits behind a router that can temporarily redirect incoming data to a lightweight receive node. Buffered studies are later forwarded to the Research PACS once service resumes.

### 4.2. Storage limitations and design trade-offs

A persistent technical challenge in ImageVU has been managing the storage and transfer of large volumes of DICOM data. Each imaging session can contain thousands of small files, with one file per two-dimensional slice. While individual files are small (typically 128–256 kilobytes), the sheer volume leads to performance bottlenecks. When we initially relied on network-based storage for the Research PACS, the system experienced frequent slowdowns and disconnections. These issues occurred despite modest data throughput, largely due to the cumulative latency of handling many small files over the network.

To mitigate this, we adopted a local caching system using solid-state drives in a redundant array. This setup allows rapid short-term access. After a delay of approximately one hour, session files are bundled into a single compressed BZIP2 file and transferred to long-term cloud storage. If prior versions of the session exist, they are retrieved and merged before upload to prevent duplication and ensure metadata consistency.

Another issue arose at the file system level. We found that performance degraded sharply when more than 5000–10,000 files were stored in a single directory, especially on standard Linux distributions. This led us to reorganize our staging directories into structured subfolders with consistent naming schemes, which significantly improved performance and stability.

Additionally, maintaining separate identified and de-identified imaging archives was deemed cost-prohibitive, leading to a single identified archive in the Research PACS, with de-identification applied on demand. For the small subset of images (approximately 1%) requiring manual de-identification, those are either processed and stored separately or excluded from the de-identified dataset if not feasible.

### 4.3. Metadata limitations and curation strategies

A persistent barrier in research imaging workflows is the mismatch between researchers’ expectations for metadata utility and the practical limitations of clinical imaging systems. Imaging science does not require in-depth knowledge of storage formats such as DICOM or the internal operations of PACS, which can lead to incorrect assumptions about how easily imaging studies can be searched and filtered.

For example, the DICOM tag BodyPartExamined is often assumed to accurately reflect the anatomical region imaged. In practice, this tag is inconsistently populated and frequently inaccurate—sometimes copied from outdated protocols or left blank entirely. Similarly, the StudyDescription field, while ubiquitous, is free-text and highly variable. It may include anything from specific protocol names to room numbers or site identifiers, making it unreliable for automated filtering or categorization.

Beyond metadata inconsistencies, architectural limitations of commercial PACS systems further hinder curation. Most systems support only minimal DICOM C-FIND operations, typically returning only high-level identifiers such as patient ID, accession number, study date, and StudyDescription. As a result, researchers often need to retrieve large numbers of studies before determining which are relevant, increasing both time and cost, and raising barriers for pilot studies.

By duplicating the clinical imaging archive into a dedicated research PACS, ImageVU enables broader and more flexible metadata indexing. Study-level metadata can be extracted and organized into portable formats, such as relational SQL databases, allowing researchers to perform rapid, fine-grained curation without depending on the constraints of clinical systems.

While ImageVU currently utilizes DIMSE-based DICOM services for data retrieval and communication, future work may explore adopting DICOMweb [32] standards to improve interoperability, enable modern web-based access, and facilitate potential cross-institutional collaborations.

### 4.4. Balancing flexible access with privacy requirements

Initial surveys of intended use cases indicated that most researchers preferred de-identified imaging data to enable linkage with the SD and BioVU. Based on this feedback, the initial ImageVU prototype was designed as a fully de-identified archive. However, as usage expanded, several projects required access to imaging linked to consented patients or other identified datasets. Additionally, de-identification requirements varied widely across studies, making it infeasible to support a single, static de-identified dataset.

This architecture of ImageVU reflects a trade-off between storage and computational cost. The cost of sustaining both the Research PACS and cloud-based storage is a significant consideration. However, this dedicated architecture is essential to prevent disruptions to clinical care while providing scalable research access. The long-term value of this system lies in its ability to support imaging-based translational research that would otherwise be infeasible. Cost projections can be estimated using tools such as the Google Cloud Storage Pricing Calculator.^2^ While on-demand de-identification increases processing demands, it significantly reduces long-term storage overhead. In practice, most research projects access only a small subset of the imaging archive, making this model cost-efficient and scalable. This approach remains effective even as study sizes increase, particularly for deep learning applications that require large datasets [33].

More recently, there has been growing interest in centralized processing workflows, in which investigators run algorithms directly against the core imaging archive. The current ImageVU architecture supports this model using scalable cloud infrastructure that enables parallelized de-identification and data access under institutional security policies. Advanced users may instantiate custom compute environments within the institutional HPC datacenter, supporting scalable analysis workflows under centralized governance.

### 4.5. Study limitations and considerations for adoption

A core principle of the VICTR model is empowering researchers by reducing barriers to data access. This is accomplished through multiple pathways, including a non-profit, fee-for-service data access core (IDASC); direct, cloud-based access with researcher-funded compute resources; and free, self-service, point-and-click interfaces such as RD Discover, SD Record Counter, and SD Discover.

Currently, ImageVU metadata are only queryable only through RD Discover, which accesses the identified metadata layer (ImageVU RD). The interface allows users to define study sets using imaging-specific criteria alongside clinical variables such as demographics, labs, medications, conditions, and procedures. Once a cohort is saved, researchers can download subject-level metadata, review available studies, and request specific series. Selected images can then be retrieved (and de-identified if needed) through billable IDASC support. Projects needing only metadata can complete the entire workflow within RD Discover at no cost.

To support early feasibility assessments, SD Record Counter enables cohort estimation without IRB approval. Any user with a valid VUNetID can perform basic queries across the clinical data warehouse and save cohorts, which can then be exported to SD Discover for deeper exploration within the de-identified SD. While de-identified imaging metadata is regularly integrated into ImageVU SD, it is not yet exposed in Record Counter or SD Discover. The VICTR Data Applications team is working to enable imaging metadata support across these tools to broaden metadata-driven cohort discovery within the de-identified data ecosystem.

ImageVU shares conceptual similarities with the OMOP Medical Imaging Common Data Model (MI-CDM [34] and OHDSI tools [35] in supporting imaging data reuse and cohort discovery. However, ImageVU is currently focused on institutional-scale, real-time imaging access tightly integrated with Vanderbilt’s local infrastructure, whereas OMOP MI-CDM and OHDSI prioritize cross-institutional standardization and federated analytics. Unlike the OMOP-based systems, ImageVU does not yet employ a common data model, but future work may explore alignment to improve interoperability.

## 5. Conclusion

Secondary use of clinical data plays a pivotal role in advancing research and healthcare innovation at academic medical centers. At VUMC, the development of ImageVU—a dedicated research imaging infrastructure—has substantially improved imaging data accessibility, streamlined cohort discovery, and supported secure, regulatory-compliant research workflows.

By integrating with the RD and SD, ImageVU enables metadata-driven imaging discovery alongside clinical and genomic data, supporting a range of use cases from feasibility analysis to high-throughput image processing. The platform’s modular design, cloud-based delivery, and compliance-aware architecture have reduced access barriers while preserving data integrity and privacy.

Beyond its local impact, ImageVU offers a replicable model for institutions seeking to develop secondary use imaging infrastructures. The lessons learned in its design and deployment provide a foundation for advancing multi-modal research, supporting precision medicine initiatives, and enabling scalable machine learning applications in medical imaging.

## Figures and Tables

**Table 1:** Statement of significance. Summary of the contribution of this work.

Problem or issue	Radiological imaging data are growing rapidly in clinical systems, but scalable, secure, and compliant reuse for research remains a persistent challenge.
What is already known	Secondary use of structured EHR and genomic data is well established, but imaging data often remain siloed due to technical, operational, and governance barriers.
What this paper adds	This paper introduces ImageVU, a research imaging infrastructure that supports metadata-driven cohort discovery, enables on-demand de-identification, and integrates local and cloud-based tools to streamline image access and reuse.
Who would benefit	Imaging researchers, informatics leaders, and clinical data scientists seeking scalable infrastructure for regulatory-compliant reuse of radiological imaging in translational research.

**Table 2:** Metadata field descriptions for ImageVU study table design.

Field name	Description
PK	Primary key
PERSON_ID	Patient ID (primary identifier)
STUDY_IUID	Unique study identifier
STUDY_ID	User or equipment-generated study ID
STUDY_DATETIME	Study acquisition date and time
ACCESSION_NO	RIS-generated accession number
REF_PHYSICIAN	Referring physician's name
REF_PHYS_FN_SX	Field dependent on ordering system
REF_PHYS_GN_SX	Field dependent on ordering system
REF_PHYS_I_NAME	Empty
REF_PHYS_P_NAME	Empty
STUDY_DESC	Study description
STUDY_STATUS_ID	Study status
MODS_IN_STUDY	Modalities included in the study
CUIDS_IN_STUDY	Class UIDs included in the study
NUM_SERIES	Number of series in the study
NUM_INSTANCES	Number of instances in the study
FILESET_IUID	Empty
FILESET_ID	Empty
CHECKED_TIME	Empty
CREATED_TIME	Study creation timestamp in ImageVU
UPDATED_TIME	Timestamp of last update to the study
INSERTED_TIMESTAMP	Initial input timestamp in ImageVU

**Table 3:** Metadata field descriptions for ImageVU Cloud Storage study table design.

Field	Description
STUDY_PK	Study primary key
PERSON_ID	PERSON_ID from the PERSON table
ACCESSION_NO	Study accession ID
STUDY_IUID	Unique study ID
STUDY_DESC	Study description
STUDY_DATE	Study acquisition date
STUDY_TIME	Study acquisition time

**Table 4:** Summary of open-source tools used in ImageVU.

Tool	Purpose
CentOS	Operating system for research servers
DCM4CHEE	Research PACS and DICOM management
Clinical Trial Processor (CTP)	De-identification processing
PostgreSQL	Metadata indexing (integrated with DCM4CHEE)
Pydicom	DICOM header processing and manipulation
Bash	Automation scripting
Slurm	Job scheduling for batch processing
Custom Python tools	DICOM manipulation and CTP inconsistency fixes
Pybossa	Custom manual review and QA platform

## Significance

The rapid growth of clinically acquired imaging data presents a transformative opportunity for secondary use in biomedical research, yet challenges related to scale, security, regulatory compliance, and data integration persist. ImageVU addresses these challenges by providing a dedicated, institutionally governed research imaging infrastructure that enables metadata-driven cohort discovery, secure access to both identified and de-identified imaging repositories, and support for automated image processing pipelines. By aligning with institutional informatics frameworks and leveraging cloud-native technologies, ImageVU enhances the efficiency, flexibility, and compliance of imaging data reuse. This positions the platform as a critical enabler of translational research, facilitating the integration of imaging into large-scale biomedical investigations while ensuring patient privacy and research integrity.

## Footnotes

1 Sectra AB, Linköping, Sweden.

2 https://cloud.google.com/products/calculator.
